# Genomic and Meiotic Changes Accompanying Polyploidization

**DOI:** 10.3390/plants11010125

**Published:** 2022-01-03

**Authors:** Francesco Blasio, Pilar Prieto, Mónica Pradillo, Tomás Naranjo

**Affiliations:** 1 Departamento de Genética, Fisiología y Microbiología, Facultad de Biología, Universidad Complutense de Madrid, 28040 Madrid, Spain; franblas@ucm.es (F.B.); pradillo@bio.ucm.es (M.P.); 2 Plant Breeding Department, Institute for Sustainable Agriculture, Agencia Estatal Consejo Superior de Investigaciones Científicas (CSIC), Alameda del Obispo s/n, Apartado 4048, 14080 Cordova, Spain; pilar.prieto@ias.csic.es

**Keywords:** allopolyploidy, interspecific hybridization, unreduced gametes, cytological diploidization, genomic changes

## Abstract

Hybridization and polyploidy have been considered as significant evolutionary forces in adaptation and speciation, especially among plants. Interspecific gene flow generates novel genetic variants adaptable to different environments, but it is also a gene introgression mechanism in crops to increase their agronomical yield. An estimate of 9% of interspecific hybridization has been reported although the frequency varies among taxa. Homoploid hybrid speciation is rare compared to allopolyploidy. Chromosome doubling after hybridization is the result of cellular defects produced mainly during meiosis. Unreduced gametes, which are formed at an average frequency of 2.52% across species, are the result of altered spindle organization or orientation, disturbed kinetochore functioning, abnormal cytokinesis, or loss of any meiotic division. Meiotic changes and their genetic basis, leading to the cytological diploidization of allopolyploids, are just beginning to be understood especially in wheat. However, the nature and mode of action of homoeologous recombination suppressor genes are poorly understood in other allopolyploids. The merger of two independent genomes causes a deep modification of their architecture, gene expression, and molecular interactions leading to the phenotype. We provide an overview of genomic changes and transcriptomic modifications that particularly occur at the early stages of allopolyploid formation.

## 1. Introduction

Polyploidy, defined as the presence of three or more complete sets of chromosomes in a cell or organism, is an important feature of genome evolution in many eukaryote taxa. Polyploids have been documented in yeasts, insects, and vertebrates [1] but polyploidy is pervasive and especially prominent in the evolutionary history of plants, with both recent and ancient events occurring particularly in lineages such as the Angiosperms [2]. Polyploids are usually classified according to their mode of origin as autopolyploids, those having three of more genomes of a given species, and allopolyploids, which originated after interspecific hybridization followed by chromosome doubling [3].

Early cytogeneticists assumed the pattern of chromosome pairing at the first meiotic division as a reliable criterion to identify homologous and homoeologous relationships between the chromosome sets of a polyploid organism. The frequency of multivalents was used as a cytological parameter to distinguish between auto- and allopolyploids [4]. A high level of multivalent pairing at metaphase I suggests homology between chromosome sets and hence autopolyploidy. In contrast, a preferential formation of bivalents likely results from the presence of non-homologous (homoeologous) parental chromosome sets, hence indicating allopolyploidy. Consistent with this notion, a survey consisting of 171 reports on neopolyploids yielded a higher frequency of multivalents at diakinesis and metaphase I in autopolyploids (28.8%) than in allopolyploids (8.3%) [5]. Such multivalent frequencies suggested partial cytological diploidization of many autopolyploids and complete cytological diploidization of most allopolyploids. However, differentiation between auto- and allopolyploids is not absolute, since almost exclusive bivalent formation was observed in some autopolyploids while some allopolyploids show multivalent formation. This variable pairing pattern is more consistent with a gradient of divergence between the genomes concurring in polyploids than with a strict differential behavior between auto- and allopolyploids.

Since the Modern Synthesis, allopolyploidy has been considered for decades to be more prevalent than autopolyploidy in the evolution of Angiosperms [6]. This trend continues today, but it is now recognized that both are extremely important in nature [2,3]. Soltis and coworkers [7] propose two main reasons for the widespread omission of autopolyploidy as a significant speciation mechanism: (i) autopolyploidy was traditionally considered extremely rare in nature, (ii) many of plant systematists adhered to a species concept based on morphological features, in which diploids and polyploids were considered as different cytotypes of a single species. In contrast, Lewis [1] proposed a significant contribution of autopolyploidy to plant speciation, especially among herbaceous perennial taxa. Further studies support that autopolyploids are frequently, though they may be lost or remain undetected [2]. Ramsey and Schemske [3] estimated a rate of autotetraploid formation of 10^−5^ similar to the genic mutation rate, which implies that allotetraploid formation can only reach such a incidence after a high frequency of interspecific hybridization (0.2% for selfing taxa, 2.7% for outcrossing taxa). They concluded that autopolyploids may often be formed at a higher frequency than allopolyploids.

Polyploid organisms may undergo major changes in genomic structure and phenotypic development, relative to their diploid counterparts, which provide them a broader basis for evolution. The presence of four alleles per locus in autotetraploids, or more in higher levels of ploidy, confers an increased heterozygosity to autopolyploids in comparison with that of their diploid progenitors. This fact allows the beneficial masking of deleterious genes. A high level of heterozygosity derived from the presence of homoeoalleles is also present in allopolyploids, which is accompanied of additivity of the merged parental genomes as well as the occurrence of instantaneous reproductive isolation, and a tendency to suffer less inbreeding depression than diploids [8]. Phenotypic and morphological changes known to be induced by polyploidy are those associated with variation in flower number and flowering time [9], root architecture and plant structure, or alterations in plant physiology such as abiotic stress tolerance [10].

The advent of the new genomic tools has led to the development of a number of molecular and genomic studies focused on the rapid changes induced by allopolyploidy, which have been revealed to occur at the genetic and epigenetic level, together with major alterations in the transcriptional landscape [11,12]. Allopolyploid formation requires the adaptation of two nuclear genomes within a single cytoplasm, which may involve programmed genetic and epigenetic changes during the initial generations following genome fusion. Early generations of synthetic allopolyploids show rapid and extensive restructuring of the merged genomes, including chromosome rearrangements and changes in the chromosome number [13,14,15,16,17,18] as well as epigenetic modifications, such as, transposon activation, chromatin modifications and altered methylation patterning [19,20,21,22,23,24]. In this first phase of allopolyploid evolution, the conflict between the merged genomes modifies the gene expression profile, which is often associated with phenotypic modifications of the new polyploid plants [25,26,27,28]. However, allopolyploids of recent origin commonly display phenotypic instability, low fertility and low embryonic viability [22], but their persistence accompanied of successful genetic readjustments may lead to stable genomic variants that express fertile and well adapted phenotypes on an evolutionary time scale of hundreds or thousands of generations [29]. This second phase of genome evolution includes either sub- or neofunctionalization of duplicated genes to form novel genetic functions and gene complex [30,31,32,33,34,35,36] or the loss of redundant copies [37]. Reduction of genomic redundancy converts the polyploid organism into a diploid one, which is often referred to as genetic diploidization. Thus, genetic diploidization is considered an evolutionary process of a wider time scale than that of the cytological diploidization which happens at the primitive allopolyploid stage and concerns genetic systems involved in control of meiotic pairing [38,39].

The evolutionary history of seed plants and Angiosperms is full of Whole Genome Doubling (WGD) events [40,41] with each subsequent polyploidy superimposed on the genomic remnants surviving from earlier rounds of polyploidy. Genomic complexity of modern Angiosperm genomes ranges from those that underwent few polyploidization events (e.g., *Amborella*, *Allium*, *Olea*, *Theobroma*) to others that reflect as many as 128 (*Saccharum*), 144 (*Gossypium*), and even 288 (*Brassica*) genomic multiplication processes [42]. Recurrent polyploidization is usually accompanied of chromosome rearrangements and reduction of chromosome number as well as a large-scale loss of duplicated genes and repetitive sequences. Such genome reorganizations, while harboring the vestigial marks of ancient polyploidy events, lead to chromosome constitutions that facilitate a cytological diploid-like behavior of the extant species [42].

Advances in understanding the extent of polyploidy in plant speciation, driven largely by the explosive development of genomic tools, have occurred in parallel with a number of reports showing evidence of mitotic and meiotic cell division alterations involved in the formation of polyploids [43]. On the other hand, recent identification of genes controlling the diploid-like meiotic behavior of allopolyploids such as wheat or *Brassica* will contribute to understand their origin and their mode of action [38,39]. In this review, we will discuss the cytological and molecular mechanisms that underlay the pathways leading to polyploids formation, including interspecific hybridization, chromosome doubling, cytological diploidization and genetic and epigenetic changes produced at the onset of allopolyploidy.

## 2. Interspecific Hybridization

Plant hybridization has been important to humans since the commencement of domestication of plants and animals during the Neolithic era. In the mid of the eighteenth century, hybridization was considered as a reproduction mode that could yield sterile plants with intermediate phenotype [44,45,46]. In the beginning of the nineteenth century, hybridization was largely used as a source of variation for plants of agronomical or ornamental importance. Its main biological relevance relied on its use as a tool to prove if two different plants deserve the species status [44]. When the hybrid obtained in a cross between two different plants was sterile such plants were considered to belong to different species while, if the offspring was fertile, they were considered as varieties of the same species. However, different naturalists challenged this idea in the second half of the nineteenth century [44]. The Mendel’s work on plant hybridization [47] and its rediscovery by de Vries, Correns and Tschermak in 1900 [48,49,50] established the path to reveal the hereditarily determined plant diversity, which, in fact, led to the emergence of genetics. The invariable offspring of homozygotes formulated in the Mendelian principles led Lotsy [51] to propose that hybridization has played a determinant evolutionary role assuming that a new combination of traits arises only by crossing. Wingë [52] proposed that new and stable species emerge by the duplication of the chromosome number of an interspecific hybrid (i.e., allopolyploidy). Evidence supporting this hypothesis was quickly reported in different plant species [53,54]. Müntzing [55] proposed a second mechanism in which hybridization may produce new and stable species. He postulated that later generation hybrids could, by chance, lead to new combinations of parental chromosomes and alleles that were homozygous for a unique combination of chromosomal sterility factors. The new hybrid population would be fertile, stable, and at the same ploidy level as its parents but partially isolated from its progenitors due to chromosome differentiation [55,56].

The formation of interspecific hybrids may be conditioned by an important aspect of the biology of the organisms, the breeding system. In plant species the mode of reproduction is sexual or asexual. Sexual reproduction takes place by self-cross, outcross or mixed. The type of sex system (hermaphroditism, monoecy or dioecy) contributes to reinforce the mode of sexual reproduction. While dioecy, and to a large extent monoecy, determine outcross reproduction, hermaphroditism ensures exclusive or dominant self-crossing. Breeding system changes along evolution even between closely related species. All changes do not occur with the same frequency, that is, the outcrossing system, which appears mainly in the ancestral lineages, is often lost and evolves to inbreeding while the transition of highly inbreeding species to outcrossing is rare [57].

Interspecific hybridization requires some rate of outcrossing of the concerned species. Outcrossing rates vary among different taxa. Most Angiosperms (87.5% [58]) are pollinated by insects and other animals and show intermediate outcrossing rates while wind-pollination species, which are mainly found among Gymnosperms and few Angiosperms families such as Poaceae, show a bimodal outcrossing-inbreeding species distribution [57,58,59].

Given the abundance of animal-pollinated species, pollinators often alternate visits to flowers of plant species flowering at the same time and within the flight pattern, which causes interspecific pollen transference. Hybridization between a given pair of species is usually asymmetric in that one species may act preferentially as male or female [60]. Effective hybridization requires a transition through several steps, foreign pollen arrival on a stigma, which generates competitive interactions with conspecific grains for adhesion and germination. Pollen must germinate and form the pollen tube, which is more likely between close relatives, as they may have similar pollen-pistil compatibility. Finally, sperm should be released to fertilize the egg. 

Competitive interactions between conspecific and heterospecific pollen grains have a negative impact on fertility whereby Angiosperms evolved multiple prezygotic and postzygotic barriers to minimize the effect of fitness reduction. Prezygotic barriers impede hybridization and include prepollination isolation and gametic selection [61]. Hybrid sterility, caused either by gene incompatibilities or chromosome rearrangements, is a common form of postzygotic reproductive isolation in plants [62]. Prepollination isolation prevents the anticipated arrival of competitive foreign pollen grains on a stigma. These barriers include adaptations such as flowering asynchrony either during the day or along the year, or divergence in floral traits conditioning the pollinator preference or the mechanical interaction between flower and pollinator during visits. Various forms of gametic isolation, including stigma incompatibility and suppression of pollen tube growth, counteract foreign pollen germination deposited on a stigma. Modifications of the structure or chemical composition of stigma as well as factors controlling pollen recognition and self-incompatibility may contribute to increase incompatibility with foreign pollen. Self-incompatibility and interspecific incompatibility show similarities in the molecular mechanisms controlling the pollen-pistil interactions, since pollen of self-compatible species deposited on stigma of self-incompatible relatives is rejected but not in the reciprocal hybridization [63]. Pollen deposited on the stigma of a distantly related species usually fails to germinate, but this is not the case between closely related species. In such cases, divergence between species in pollen tube performance in the style may cause either a more disruptive development of the foreign pollen tube or mismatches in the size of both structures, which contribute to avoid interspecific hybridization [61]. An additional evolutionary strategy to avoid interspecific hybridization involves a shift in the mating system toward earlier self-pollination, which reduces the opportunity of fertilization after the arrival of foreign pollen to stigma [61].

Despite genetic isolation barriers arisen in the evolution, interspecific hybridization is a relatively frequent biological phenomenon. Whitney and coworkers studied the pattern of hybridization in a sample of approximately 10% of 352,000 Angiosperm species [64]. They investigated 37,000 species included in 3212 genera of 282 families from eight regional floras covering parts of North America and Australia, continental Europe and two island groups (British Isles and Hawaii). Interspecific hybrids occurred with a frequency of 0.09 hybrids per non hybrid species. Both families and genera had different hybridization propensity, but a given group usually showed the same hybridization pattern across regions, which suggests that genetic constitution of each group is more relevant than environmental conditions. This hybridization frequency based on floristic surveys was probably underestimated, and more accurate estimates are expected to be obtained through implementation of genomic tools. Natural hybridization has been reported in 22 of the 25 most important crops of the world and is also common among invasive species [65].

Interspecific hybridization may represent a source of phenotypic innovation [62]. Although interspecific hybrids usually show a low fertility, the offspring obtained allows to extend hybridization to further generations, which may display transgressive trait variation, that is, a gain or a loss of valuable traits with respect to the parental species. On the other hand, recurrent backcrosses of the hybrids with one of the parental species give rise to introgression of alien genes in any of the parental species and represent a common way of gene flow between species in hybrid zones [62].

While interspecific hybridization and subsequent WGD (allopolyploidy) represents a common mode of speciation in plants, homoploid hybrid speciation is rare. Some hybrid lineages found in *Helianthus*, and probably in other genera, have achieved reproductive isolation without variation of the ploidy level [62]. Darlington [66] proposed an inverse relationship between the fertility of an interspecific hybrid and the fertility of the allopolyploid generated from the homoploid hybrid by doubling its chromosome number. He argued that homoploid hybrids between closely related species will show a high level of meiotic pairing and fertility, while the fertility of the corresponding allopolyploids will be reduced because of uneven segregation of chromosomes from multivalents involving both homologues (equivalent chromosomes from the same genome) and homoeologues (equivalent chromosomes from related genomes). In contrast, homoploid hybrids between distant species will be sterile due to chromosome pairing failure, but allopolyploids will be fertile due to preferential formation of homologous bivalents at meiosis. Comparison of the mean genetic distance between the parental species of homohybrids and allopolyploids in different taxa led to the conclusion that polyploid formation occurs at random regardless the level of phylogenetic divergence between the parental species while homoploid hybrids tend to be formed among progenitors closely related [67].

## 3. Mechanisms and Frequency of WGD in Plants

### 3.1. Pathways for WGD in Plants

The occurrence of WGD in interspecific hybrids contributes to stabilize their meiotic behavior strongly disrupted when homoeologous chromosomes fail to pair. Meiotic pairing regularization ensures a high degree of fertility of new allopolyploids, whose genomic integrity may persist through generations. Thus, upon hybridization, WGD drives allopolyploid induction and speciation. However, our understanding of the processes affecting the initial formation of polyploids within diploid populations is one of unexplored issues of polyploidy evolution [68]. Cytological alterations reported in the last two decades, such as meiotic non-reduction giving rise to 2n gametes and somatic doubling, have been implicated in the formation of individuals with changed ploidy level [43]. Union of unreduced gametes, rather than somatic doubling, has been considered as the most likely method of polyploid formation in plants [69,70].

Meiotic non-reduction or meiotic restitution is the outcome of cellular alterations in any of the two meiotic divisions that switch meiosis into a mitotic-like division, generating two diploid spores out of a diploid mother cell instead four haploid spores. An autotetraploid individual can be originated either in one-step process consisting in the fusion of unreduced egg and unreduced sperm or in a two-steps process that involves formation of a triploid intermediate after fusion of an unreduced gamete with a normal haploid gamete, followed of self-fertilization of the triploid or crossing with a diploid [3]. Most gametes produced by triploids are not functional because of their aneuploid constitution, but triploids generate a low number of euploid gametes (×, 2× and even 3×) which are involved in the formation of the autotetraploid. Allotetraploids can be formed in one-step process from interspecific hybrids, but the triploid bridge is also possible as triploid interspecific hybrids have been observed in different taxa [3].

### 3.2. Cellular Mechanisms, and Their Genetic Control, Causing Plants Meiotic Restitution

Cellular defects giving rise to meiotic restitution have been classified in two main groups, namely First Division Restitution (FDR) and Second Division Restitution (SDR), which yield 2n gametes genetically equivalent to those produced by a loss of the first meiotic division or the second meiotic division, respectively [43,71,72,73]. In the absence of recombination, the FDR-type is comparable to a mitotic division and 2n gametes retain the parental genome constitution, but when one crossover is formed between two homologues the parental constitution is retained in only two of the four chromatids. Unreduced gametes produced in the SDR-type contain the two sister chromatids of the same parental or recombinant chromosome. Cellular defects causing meiotic restitution have been classified in three main types: alterations of meiotic spindle dynamics, disturbed formation of meiotic cell plate, and omission of any of the two meiotic divisions [43,71,72,73].

Spindle dynamics in any of the two meiotic divisions may be altered by structural defects in microtubules nucleation, spindle organization and orientation, and kinetochore functioning, which usually generate unbalanced chromosome segregation and aneuploidy, but occasionally induce a meiotic restitution event. Formation of curved spindle and disturbed attachment of microtubules to kinetochores impede chromosome segregation and block cell plate formation in the first meiotic division of interspecific wheat-rye hybrids, yielding a restituted nucleus, which undergoes the second meiotic division to form unreduced gametes [74]. Other alterations in the spindle organization disturb its bipolar orientation causing monopolar, multipolar or apolar arrangements of microtubules. Meiocytes with monopolar spindle, in interspecific hybrids of Triticeae, or apolar spindle, in maize meiotic mutants, omit chromosome segregation at anaphase I yielding unreduced gametes [75,76]. Abnormal spindle geometry in the second meiotic division has been documented as a source of unreduced gametes formation in dicotyledons microsporogenesis [71]. Proper chromosome number reduction is produced when the two spindles adopt a perpendicular orientation in the second meiotic division, followed by cytokinesis to yield the four microspores. Disturbed arrangements such as fused spindles and parallel spindles generate a dyad with 2n nuclei while a tripolar spindle produce a triad containing two n and one 2n nuclei [77,78,79,80]. However, fused, parallel and tripolar spindles may appear in the same flower and probably represent different phenotypic expressions of the same cellular defect [79,80,81,82].

Alterations of spindle dynamics are under strong genetic control. Up to date, a list of 23 genes of *Arabidopsis*, whose mutations cause meiotic restitution, have been reported [83]. Instances of *Arabidopsis* genes with mutations that induce the formation of unreduced 2n microspores through alteration of the second meiotic division spindles arrangement are *AtPS1*, *JASON* and *AFH14* [79,80,84]. Protein AFH14 is involved in the control of cell division trough interactions with microtubules and microfilaments. The mode of action of *AtPS1* on spindle organization is unknown. The AtPS1 protein has been suggested to play its regulatory function via RNA decay [80]. The JASON protein positively regulates the *AtPS1* expression, which suggests that JASON controls the organization of the second meiotic division spindles through AtPS1 [80]. On the other hand, the origin of tetraploid potato cultivars and related wild species has been attributed to a high frequency of the *ps* (parallel spindles) allele, which induces the formation of 2n gametes [85,86]. In carnation, *Dianthus caryophyllus*, the *DcPS1* (*Dianthus caryophyllus Parallel Spindle 1*) gene, which encodes a protein with domains suggesting a regulatory function, induces the formation of unreduced gametes through alteration of spindle orientation in the male second meiotic division [87].

Abnormal cytokinesis, either in the first or in the second meiotic division, represents another major mode of unreduced gametes production both in microsporogenesis and macrosporogenesis. In monocotyledons, cytokinesis occurs at the end of the two divisions of both male and female meiosis, but, in the spermatocytes of most dicotyledons, cytokinesis takes place at the end of the second division. Premature cytokinesis in the first meiotic division of microsporocytes although reported in some dicotyledons species is not a relevant mechanism of meiotic restitution [71,78]. Incomplete or no cell plate formation at any meiotic division has been observed in different species and represents an important mechanism of FDR- or SDR-type unreduced gametes [88,89,90,91,92,93]. Cellular defects such as disturbed microtubule array biogenesis or reduction of microtubules stability [94,95,96,97] anomalous transport of cell wall material, disturbed fusion of membrane vesicles [98] and reduced deposition of callose [99] may prevent cell plate formation in meiotic cells. The chromatin regulator Male Meiocyte Death 1 (MMD1/DUET), a PHD-finger protein that binds with H3K4methylation sites, is involved in the control of multiple processes in *Arabidopsis* male meiosis. A hypomorphic *mmd1/duet* mutant allele causes defects in microtubule organization and cytokinesis, which leads to meiotic restitution [83]. Disturbed spindle elongation or orientation may also induce cytokinesis alterations. This is the case of potato and *Populus* meiocytes, where disturbed spindle orientation causes defects in interzonal radial microtubules array formation followed of cell plate formation failure and unreduced gametes [78,100].

Loss of any of the two meiotic cell divisions is another source of meiotic restitution. The absence of the first meiotic division abolishes both homologous recombination and chromosome number reduction. Chromosomes divide at anaphase II separating sister chromatids in two FDR-type nuclei with the parental genetic constitution [101,102,103,104]. This phenomenon is observed in apomictically reproducing species and is termed diplosporous apomeiosis [105,106,107,108]. In fact, the three components of apomixis, apomeiosis, parthenogenesis and functional endosperm development, are uncoupled in many crops leading to their partitioning [109]. Mutation of the *Arabidopsis* gene *DYAD/SWITCH1* (*SWI1*), a regulator of meiotic chromosome organization, causes apomeiosis [102]. The transformation of meiosis into a mitotic division was also shown in the triple *osd1*/*Atrec8*/*Atspo11-1* mutant of *Arabidopsis*, called *MiMe*, where the *Atspo11-1* and *Atrec8* mutations produce sister chromatids separation at anaphase I, and the *osd1* mutation prevents the second division [103]. Sister chromatids separate in the first meiotic division while the second meiotic division is omitted. The *Dominant nonreduction4* (*Dnr4*) of maize is defective in chromatin condensation during meiosis and shows a diplosporous phenotype with frequent unreduced gametes formation [104]. *Dnr4* codes for AGO104, a member of the ARGONAUTE family proteins, which is needed for non-CG methylation of centromeric and knob-repeat DNA. Mutation in protein-coding genes, such as *Arabidopsis* protein DYAD/SWITCH1 [110], maize DMT102 and DMT103 DNA-methyltransferases [111], and APOLLO (APOmixis-Linked Locus) histidine exonuclease of *Boechera* [112], induce a complete omission of the first meiotic division and yield meiocytes that undergo equational cell division to produce 2n megaspores.

Failure of separation of sister chromatids in the second meiotic division because of delayed dissolution of centromeric cohesion yields dyads with SDR-type 2n nuclei [103,113]. Mutations in the *Arabidopsis* genes *GIGC1/OSD1* (*GIGAS CELL1/OMISSION OF SECOND DIVISION1*) and *CYCA1;2* (*A1-TYPE CYCLIN*), also called *TAM* (*TARDY ASYNCRONOUS MEIOSIS*), cause the second division blocking after normal chromosome segregation in the first division, generating dyads containing SDR-type 2n nuclei [103,113]. OSD1 is involved in the maintenance of the activity of cyclin-dependent kinases and promotes meiotic division through inhibition of the APC/C (Anaphase Promoting Control/Cyclosome) [114]. The A-type cyclin CYCA1;2 encoded by TAM is involved in regulation of cell cycle progression through the formation of a complex with CDKA;1 [115].

### 3.3. Meiotic Restitution in Interspecific Hybrids

The absence of homologous chromosomes in interspecific hybrids restrict meiotic pairing to homoeologous chromosomes, which show a variable synaptic pattern conditioned by the degree of affinity between the parental genomes. Such a meiotic irregularity affects chromosome segregation at anaphase I and causes a high frequency of non-viable gametes. However, some interspecific hybrids between Triticeae species are capable of producing small or sometimes large numbers of seeds, which usually have a duplicated chromosome number [116,117,118,119,120,121,122]. Hybrids between phylogenetically distant species, such as wheat x rye, lack meiotic recombination and form, almost exclusively, univalents at metaphase I. These univalents either show a monopolar orientation and segregate to one pole at anaphase I [75,118] or once positioned at the cell equator do not move to the poles [118], or after segregation at anaphase I undergo a centripetal migration from the poles toward the cell center at telophase I [74], yielding in all instances one restituted cell, which undergoes a normal second division. Sister kinetochores of chromosomes forming bivalents orient syntelically at the first meiotic division, but those of univalents may show either syntelic or amphitelic orientation [123]. Sister kinetochores of univalents undergoing monopolar segregation, as well as those moving from the poles towards the cell center and rejoined on the equator at telophase I, should adopt a synthelic orientation. In contrast, univalents staying at the cell equator are oriented amphitelically. This was demonstrated in a study [124] on the orientation of sister kinetochores in meiocytes of durum wheat cultivar Langdon, which orient syntelically in the bivalents formed in tetraploid plants but amphitelically in the univalents produced in polyhaploids and interspecific hybrids with *Ae. tauschii.* Persistence of sister centromere cohesion until anaphase II counteracted the tension created by amphitelic orientation and maintained univalents at the cell equator contributing to generate the restituted nucleus. Univalents formed in interspecific hybrids may also divide equationally at anaphase I. When all univalents behave in this way, the second meiotic division is lost and meiosis is converted in a single mitotic division, which is called single-division meiosis (SDM). SDM has been observed in interspecific hybrids of durum wheat and *Ae. longissima* [120,125] and in hybrids of durum and bread wheat with other *Aegilops* species or rye [126,127]. Both FDR and SDM meiotic restitution types coexist in the same hybrid [125,126,127]. Failure of synapsis in polyhaploids and interspecific hybrids was considered essential in determining the type of centromere orientation of chromosomes at metaphase I and anaphase I. Cytomixis, a migration of cytoplasmatic or nuclear materials between adjacent cells, has also been reported as source of meiotic restitution in hybrids between the parental species of peanut (*Arachis hypogea*, L) [128].

Jauhar and coworkers [118] suggested that in interspecific hybrids, meiotic restitution and its frequency largely depend on the presence of univalents caused by the lack of homologous partner rather than genetic defects. Consistent with this assumption is the behavior of ABDD hybrids obtained in the cross of *T. turgidum* (AABB) × tetraploid *Ae. tauschii* (DDDD), which do not undergo meiotic restitution while the ABD *T. turgidum* × *Ae. tauschii* hybrids do [129]. A similar conclusion was inferred from the occurrence of meiotic restitution induced by the formation of univalents in durum wheat haploids, which is absent in haploids of the 5D-5B substitution line where homoeologous bivalents are formed [118]. Similarly, asynapsis induced by low temperature in nulli 5D-tetra 5B plants of hexaploid wheat Chinese Spring generates meiotic restitution [130]. Thus, failure of synapsis more than absence of a homologous partner is responsible of meiotic restitution induced by univalents formation in hybrids. However, genetic factors were also suggested to be involved in the induction of unreduced gametes in genomic combinations involving durum wheat and *Ae. longissima* chromosomes [125]. Accordingly, genetic differences between *T. turgidum* genotypes are responsible of differences in the frequency of chromosome doubling observed in hybrids with rye or *Ae. tauschii* [131,132,133,134]. An allele causing meiotic restitution in such hybrids has been located on chromosome 4A in cultivar Langdon [135,136] and one quantitative trait locus (QTL) on chromosome 3B [137]. Genetic variation in the promotion of unreduced gametes formation through meiotic restitution in ABD hybrids was also observed in *Ae. tauschii*, which seems to be under the control of six QTLs [138]. *Ae. triuncialis* shows also genetic variation in its ability to promote chromosome doubling in hybrids with wheat [139]. Studies in haploids of wheat-rye substitution lines revealed that univalents divided equationally at anaphase I in the presence of chromosome 6R but reductionally when 2R was present [140]. Thus, these two rye chromosomes cause antagonist effects on meiotic restitution, 6R is a promoter chromosome and 2R a suppressor one of meiotic restitution. A further study confirmed these results and that chromosome 1R and 5R carry also genetic information that promotes equational division of univalents and SDM [127].

Cytological mechanisms leading to meiotic restitution in interspecific hybrids between species sharing a relatively high level of genetic affinity are similar to those reported in hybrids between more distant species, but genotypes of unreduced 2n gametes formed show usually a higher frequency of homoeologous recombination than those produced in a strict FDR-type mechanism. Bivalents and multivalents formed by homoeologous chromosomes in metaphase I, combined with separation of sister chromatids of univalents, generate dyads with unusual chromosome constitution consisting in nulli-disomies and associated translocations of homoeologues [141]. This process of unreduced gametes formation was first detected in hybrids of *Lilium* and termed indeterminate (IMR)-type of meiotic restitution [142]. Polyploids formed from such unreduced gametes show a mixture of two copies- and four copies-chromosomal regions, are called segmental allopolyploids [143], and have been detected in different taxa through cytological approaches or genome sequencing [144,145,146,147,148].

### 3.4. Stress-Induced Meiotic Restitution

Plant meiosis is extremely sensitive to environmental conditions. Abiotic stresses, such as low and high temperatures, salt stress, osmotic shock and water deficit, have a negative impact on male gamete development and cause a considerable reduction of male fertility [149,150]. Adverse conditions are also a source of meiotic restitution in species or hybrids of genera *Rosa*, *Solanum*, *Populus*, *Impatiens*, *Agave*, *Lotus*, *Ipomnoeoa*, *Capsicum*, *Triticum*, *Arabidopsis* or *Medicago* [43]. Heat stress or short period of cold induce meiotic restitution through irregularities on spindle organization or orientation, alteration of cell cycle regulation, defects on cell wall formation, or failure on synapsis and chiasma formation [43]. A more frequent formation on unreduced gametes in extreme environments has been suggested based on the observation that polyploids are more prevalent in such conditions [151]. This assumption in association with the coincidence of a burst of ancient polyploidization events with the Cretaceous-Paleogene extinction, occurred 66 million years ago, suggesting that polyploid establishment is promoted during times of environmental stress [152,153].

### 3.5. Premeiotic and Postmeiotic WGD in Plants

Apart from meiotic non-reduction, diploid gametes can also be generated by premeiotic or postmeiotic WGD [154]. Premeiotic WGD may be the result of two different cytological abnormalities, syncyte formation and cytomixis. The syncytium is formed because of defects in cell wall construction and, when it is followed of nuclear fusion, polyploid cells arise, which generate diploid gametes. This was observed in the tomato mutant *pmcd1* (*pre-meiotic cytokinesis defect 1*), tetraploid meiocytes originate from nuclear fusion events in syncytial premeiotic germ line cells, ectopically generated by alterations in cell wall formation [155]. Cytomixis leading to WGD was reported in *Dactylis glomerata* [156]. Migration of chromosomes through cytomictic channels was observed during the entire first meiotic division from pachytene to telophase I. The number of bivalents in some meiocytes at diakinesis indicated the formation of some polyploid cells, which generate unreduced gametes. Postmeiotic chromosome doubling occurs by defects in cytokinesis after the second meiotic division. Cold treatment disturbs the phragmoplast construction by destabilization of radial microtubule arrays at the tetrad stage in *Arabidopsis* [91]. Defects in microtubule organization cause also errors in cell wall formation in the *tetraspore* (*tes*)/*stud* mutant of *A. thaliana*, the absence of cytokinesis generates a monad with four nuclei, some of which may be fused [157]. Postmeiotic cytokinesis is regulated by the mitogen-activated protein kinase (MAPK) signaling pathway. *TES/STUD/AtNACK2*, *MKK6/ANQ1*, and *MPK4* are the three main components of this MAPK signaling cascade and mutations of these genes cause failure of microspore mother cell cytokinesis, which results in over-size mature multinucleate pollen grains [95].

### 3.6. Frequency of Unreduced Gametes Formation in Natural Plant Populations

The importance of 2n gametes for polyploid evolution raises the question of how frequent and variable 2n gametes are in natural populations. The estimation of the frequency and variation of unreduced gametes in natural populations has been rather limited. Most reports were based in a few number of populations in a small number of species and suggested a low frequency of 2n gametes [158,159,160,161,162,163]. The most relevant study to compare the frequency and variation of 2n gametes formation within and among taxa was carried out by Kreiner and coworkers in 60 populations from 24 species of Brassicaceae [164]. The presence of unreduced male gametes was detected by flow cytometry, which establishes the level of ploidy by quantification of the DNA content of pollen nuclei. Variation of 2n gametes production was assessed among species, among populations within species, and among individuals within populations. Most of the variation in 2n gametes production was among individuals within populations. The proportion of 2n gametes per plant ranged from 0 to 85.6%, with an average frequency of 1.93% among all plants and 2.52% across species. Variation in 2n gamete production was related to reproductive system; asexual species produced significantly more 2n gametes than mixed-mating and outcrossing species. The conclusion was drawn that in situations of low selective pressure, 2n gametes can be maintained and individuals with high frequency of unreduced gametes are important to generate polyploid offspring. 

## 4. Cytological Diploidization of Allopolyploids

After WGD, multivalents at metaphase I lead to missegregation, gametic aneuploidy and low fertility [165,166]. In this landscape, natural selection should favor a diploid-like meiosis (cytological diploidization) with bivalent chromosome configurations, even though there are four or more sets of homologous/homoeologous chromosomes, to overcome the reduction in fertility derived from meiotic irregularities. As explained above, autopolyploids and allopolyploids face different meiotic challenges for balanced segregation of homologous chromosomes during diploidization [5,167]. We will address below specific examples of these situations concerning mainly allopolyploid species among monocots and dicots.

### 4.1. Monocots

#### 4.1.1. Wheat

**Figure 1:**
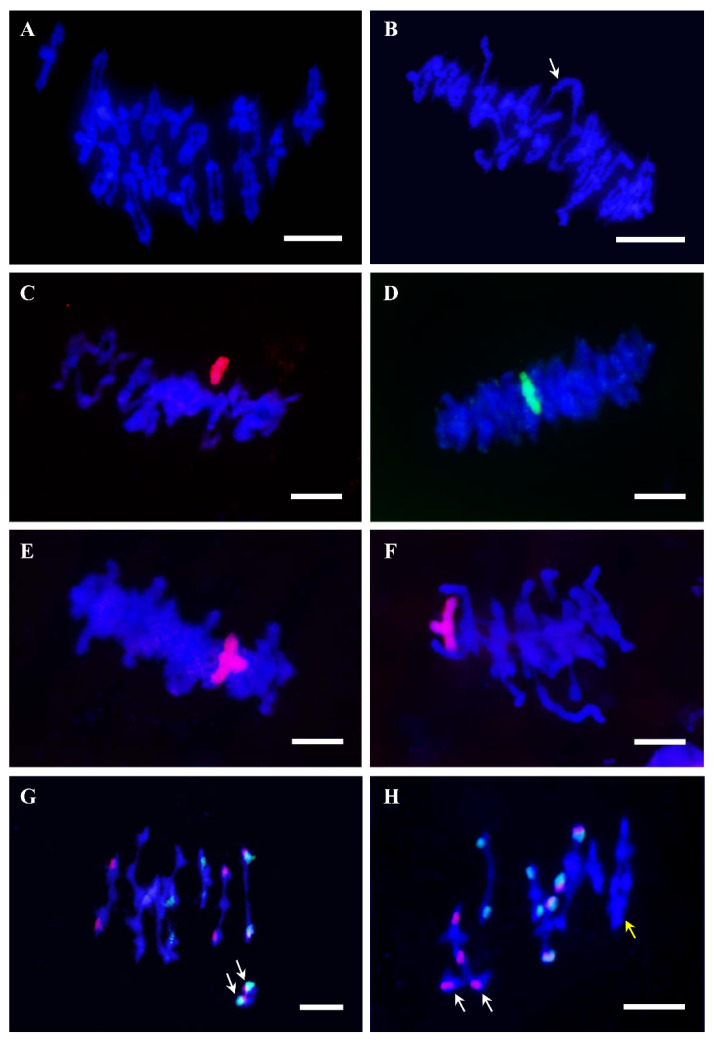
Diploid behavior during meiosis in allopolyploids and autopolyploids. (**A**,**B**) Chromosome associations at metaphase I in the presence and in the absence of the *Ph1* locus in wheat (*T. aestivum*; AABBDD). (**A**) Representative image showing regular bivalent formation in the presence of *Ph1.* (**B**) Formation of multivalents (arrow) in the absence of *Ph1*. (**C**–**F**) Fluorescence in situ hybridization showing chromosome associations at metaphase I in wheat lines carrying chromosomes from wheat related species, both in the presence and in the absence of the *Ph1* locus. Introgressed homologous chromosomes are visualized associated in disomic lines independently of the presence of the *Ph1* locus, although aberrant chromosome associations can be observed in the absence of the *Ph1* locus (**F**). (**C**) Wheat + pair 6H^v^ from *Hordeum vulgare* (red), *Ph1Ph1.* (**D**) Wheat + pair 6H^ch^ from *Hordeum chilense* (green), *Ph1Ph1.* (**E**) Wheat + pair 6P from *Agropyron cristatum* (in red), *Ph1Ph1.* (**F**) Wheat + pair 6P from *Agropyron cristatum* (in red), *ph1ph1.* (**G**,**H**) Chromosome associations at metaphase I in a natural autotetraploid line from *A. thaliana*. 45S rDNA and 5S rDNA regions are detected in green and red, respectively, to identify the chromosomes. (**G**) Metaphase I showing a pair of univalents (arrows). (**H**) Metaphase I exhibiting univalents (white arrows) and a quadrivalent (yellow arrow). Bars for (**A**–**F**): 10 µm. Bars for (**G**,**H**): 5 µm.

Bread wheat is one of the most important crops in the world. It is the only allopolyploid species in which an extensive amount of work has been carried out to study cytological diploidization and identify genes involved in the genetic control of recombination [168,169,170], particularly among homoeologous chromosomes [171,172]. This allopolyploid species, and tetraploid (pasta) wheat, arose by combining related genomes. Bread wheat (*Triticum aestivum* L., 2n = 6x = 42) comprises three subgenomes (A, B, and D) derived from three different but related diploid species. Each subgenome contains seven pairs of homologous (equivalent) chromosomes. Similarly, allotetraploid wheat (*T. turgidum* L., 2n = 4x = 28), commonly known as durum (or pasta) wheat, has also two subgenomes (A and B) with seven pairs of homologues each. Chromosomes from different subgenomes are named homoeologues since they evolved from a common ancestor and preserve a considerable degree of genetic affinity [173]. The presence of homoeologous chromosomes, which share a high degree of gene synteny and DNA sequence homology, makes the process of recognition and pairing during meiosis more complicated because each wheat chromosome needs to distinguish between its equivalent (homologue) and the similar/related (homoeologue) from the other subgenomes. Hence, despite its genome complexity, wheat behaves as diploid during meiosis (Figure 1). This means that chromosomes associate regularly in pairs of homologues to successfully recombine and segregate correctly in anaphase I. This high efficiency of chromosome associations in pairs during meiosis has a great effect on wheat fertility but, on the other hand, has a negative effect preventing pairing and recombination between wheat chromosomes and those from related species in the framework of breeding.

Numerous challenges at the cytological, genetic, and epigenetic levels were overcome to preserve fertility in the newly formed allopolyploids, particularly in annual and predominantly self-pollinated species such as wheat and wheat related species [174]. At the cytological level, the diploid-like meiotic behavior in wheat has been traditionally explained through the action of several *Ph* (*Pairing homoeologous*) genes, which restricted chromosome associations to homologous chromosomes [175,176,177]. Among these *Ph* genes, the *Ph1* locus, located on the long arm of chromosome 5B, was described as the major chromosome pairing locus in wheat [178]. Other loci such as *Ph2* located on the short arm of chromosomes 3D, or another suppressor located on the short arm of chromosome 3A, have also an effect on meiosis, although their impact is much weaker than the one of the *Ph1* locus [176,179,180].

The *Ph1* locus has been intensively studied during some decades due to its key implications in meiosis and therefore in breeding. Several hypotheses have tried to explain how *Ph1* restricts recombination to homologous chromosomes (reviewed in [38]). The *Ph1* locus was described controlling homologous chromosome pairing in bread wheat [175,181,182,183]. The presence of the *Ph1* locus affects also the dynamics of telomere bouquet formation by delaying it, what suggests that chromosomes might have more time to check potential pairing and consequently, correct homologous chromosome pairing could be facilitated [184]. Suppression of homoeologous crossovers (COs) instead of preventing chromosome associations between homoeologues has been also assessed to the *Ph1* locus [185,186,187,188,189]. 

Recently, the *ZIP4* gene has been associated to the *Ph1* phenotype. *ZIP4* was included during polyploidization in the same region of chromosome 5B and consists in one extra copy of the major new meiotic gene *ZIP4*, named *TaZIP4-B2*, that duplicated and diverged from chromosome 3B [172,186]. Hence, hexaploid wheat has four copies of *ZIP4*, one copy on chromosomes 3A, 3B and 3D, and a fourth copy inserted on chromosome 5B, which corresponds to the duplicated and diverged *TaZIP4-B2* [190].

The new finding of the *TaZIP4-B2* gene as the candidate to explain the effect of the *Ph1* locus on recombination suggests that *TaZIP4-B2* has a stronger effect in meiosis than previously explained for *ZIP4* in other model species studies [190,191]. In *Arabidopsis* and rice, *ZIP4* is only necessary for homologous CO and not for pairing and synapsis, although in yeast, *ZIP4* is needed for both CO and synapsis [191,192,193,194]. In addition, *ZIP4* can also participate as a scaffold protein that facilitates the assembly of protein complexes and promoting homologous COs [190,191,195]. Studies in wheat comparing two *TaZIP4-B2* TILLING mutants, one *TaZIP4-B2* CRISPR mutant and the Sears *ph1b* deletion mutant have revealed that all four mutants display an equivalent level of COs between homoeologous chromosomes in hybrids with the same wild relative [196]. Due to the fact that *TaZIP4-B2* TILLING and CRISPR mutants are of recent origin, the possibility that chromosome rearrangements accumulated over generations in the *ph1b* mutant could also modify the meiotic phenotype derived from the absence of the wild *Ph1* allele, seems unlikely. In addition, large-scale genome sequencing and RNA analysis have recently shown that homoeologous wheat chromosomes did not display wide gene loss or expression changes after polyploidization [197,198], suggesting that a key factor quickly evolved upon wheat polyploidization to regulate the behavior of its several genomes at the onset of meiosis, and consequently fertility is also preserved. Altogether, after several decades of studying the diploid-like behavior of polyploid wheat, the duplicated and diverged *TaZIP4-B2* copy inserted on wheat chromosome 5B seems to be the key regulator, responsible for both the suppression of homoeologous COs and the promotion of homologous pairing-synapsis phenotypes, which has been historically defined on wheat chromosome 5B. Nevertheless, the molecular mechanisms behind its phenotype remains to be elucidated.

#### 4.1.2. Wheat-Related Polyploid Species 

Among allopolyploid *Aegilops* species, regardless the degree of divergence between homoeologous genomes, the control of the diploid-like meiosis operates by means of restriction of synapsis to homologous chromosomes and suppression of chiasma formation in the infrequent homoeologous associations [199]. A similar system controlling restriction of chromosome synapsis initiation to homologous chromosomes has also been reported in allopolyploid species of *Avena* [200], *Festuca* [201], and wild forms of *T. turgidum* and *T. timopheevii* [202]. Surprisingly, the strictly disomic inheritance displayed by the cultivated wheat *T. timopheevii* is achieved though synaptonemal complex (SC) multivalents are relatively frequent during prophase I [203]. The high efficiency of the diploidizing mechanism of *Aegilops* is noticeable, at least in *Ae. ventricosa*, since it also operates in the synthetic amphiploid *Ae. ventricosa-S. cereale* despite its recent origin and the presence of rye genomes [199].

#### 4.1.3. Other Polyploid Species Included in the Poaceae Family

In the *Zea* genus, which includes allotetraploid species with 2n = 20 chromosomes, such as maize, *Zea mays* L., and teosintes, and the alloautooctoploid species *Z. perennis* (2n = 40), a paring regulator locus (*PrZ*), whose expression is suppressed by colchicine, has been recently reported [204]. Poggio and González postulated that, in *Z. perennis*, *PrZ* would affect independently the A and B maize genomes, being relevant the threshold of homology, the fidelity of pairing in each genomes and the ploidy level [204]. To the best of our knowledge, no other genes related to maize cytological diploidization have been described so far.

Little efforts have been carried out to shed some light on the diploid-like behavior of rice polyploids. The genus *Oryza* has 24 species, two of them (*O. sativa* and *O. glaberrima*) are cultivated and 22 are wild species. Among the 22 wild species, six are in the primary gene pool of *O. sativa* complex and share the A genome. Another group of 10 wild species, under the *O. officinalis* complex, includes tetraploid species with genome constitution, BBCC (*O. punctata* and *O. minuta*) or CCDD (*O. latifolia*, *O. alta* and *O. grandiglumis*). All species of this complex belong to the secondary gene pool and are cross incompatible with *O. sativa*. The other six wild species are most distantly related and highly cross incompatible to *O. sativa* and include tetraploids with genome formula HHJJ and HHKK [205]. Particularly in the *O. officinalis* complex, the largest of the *Oryza* genus, genomic relationships were found extremely complicated. For example, the BBCC tetraploid species formed independently with different parenthood in three polyploidization events [206,207,208,209]. Furthermore, three tetraploid species with CCDD genomes were assumed to be formed by one polyploidization event, where the CC genome progenitor was the maternal parent [206,209,210,211,212]. All these works revealed that the C genome seems to be the pivotal genome in all the tetraploids rice species.

Nevertheless, a *Ph1*-like system has not been identified so far in the genus *Oryza*. Recent studies have been focused on the temporal evolutionary dynamics of four polyploid genomes at both genetic and expression levels. Orthologous genomic sequences adjacent to the *DEP1* locus, a major grain yield QTL in cultivated rice, from four *Oryza* polyploids and their likely diploid genome donors or close relatives have been studied [213]. Genome dominance of this locus was not detected in the lately formed BBCC polyploid, *O. minuta*, and its short-term reactions to allopolyploidy is mainly displayed as a high fraction of homoeologous gene pairs showing imbalanced expression. In addition, an ongoing diploidization progression has been detected in this genus, suggesting that the expression divergence conducted by changes of selective restriction might plays an important function in the long-term diploidization [213].

### 4.2. Dicots

#### 4.2.1. *Brassica napus*

As it happens in allopolyploid wheats, in allohaploids (AC, 2n = 1x = 19) from oilseed rape (*Brassica napus*, AACC; 2n = 4x = 38) homoeologous chromosome pairing during meiosis is genetically controlled by a major QTL named *PrBn* for *PAIRING REGULATOR IN B. NAPUS* [17,214,215,216]. *PrBn* has an effect on the frequency but not on the distribution of chiasmata between homoeologous chromosomes [217]. In addition to this locus, in this species, other six minor QTLs have slight additive and *PrBn*-independent effects on non-homologous chromosome recombination frequency [218]. *PrBn* was identified by exploiting natural variation for high and low homoeologous recombination in *B. napus* haploids. However, all *B. napus* allotetraploid accessions display a diploid-like meiotic behavior regardless the genotype at the *PrBn* locus [214,217]. Therefore, the mode of action of *PrBn* seems to be different from that of *Ph1* in wheat. Unlike *PrBn*, no natural polymorphism has been described for *Ph1* in hexaploid wheat and, in contrast to *Ph1*, *PrBn* is not required for regular bivalent formation during meiosis in the allopolyploid *B. napus.* Since the suppression of homoeologous pairing by *PrBn* is not essential, the mechanism of meiotic stability in *Brassica* remains unclear.

Attempts have been made to identify the candidate gene, but this has not been possible, even though it has been mapped to chromosome C9 [214]. Annotated genes within the QTL region includes *RPA1C* (*REPLICATION PROTEIN A 1C*) and *MUS81* (*MMS and UV SENSITIVE 81*). RPA1C functions in double-stranded break (DSB) repair during meiosis in *Arabidopsis thaliana* [219] and MUS81 is an endonuclease involved in the formation of crossovers (COs) [220]. However, expression analyses on meiocytes isolated from the two lines used to map the *PrBn* locus, *Darmor-bzh* and *Yudal*, revealed no differences [221].

Cifuentes and coworkers [215] found that two meiotic phenotypes of *B. napus*, differing in the chiasma frequency at metaphase I, could be explained by the segregation of two alleles at *PrBn*. These alleles came from different parental *B. oleracea* (CC, 2n = 2x = 18) genotypes. In a similar study, Sheidai [222] found variability in pairing and chiasma frequency associated to different *B. napus* accessions. Mason and Batley [223] suggest that genetic control of chromosome pairing in *B. napus* could arise either by mutation in the newly formed allotetraploid or through the accumulation of minor alleles inherited from the diploid parents. Interestingly, in contrast to the regular bivalent formation in *B. napus* lines, resynthesized allotetraploids (obtained by hybridization of the parental species *B. oleracea* and *B. rapa*) display a high frequency of homoeologous bivalents and even multivalents [224]. Exploiting these differences, Higgins and coworkers [225] identified recently three QTLs that contributed to the control of homoeologous recombination. One of these QTLs, *BnaPh1* (*B. NAPUS PAIRING HOMOEOLOGOUS 1*), is the major contributor to variation of the recombination pattern. This QTL locates in a homoeologous region of that carrying *PrBn*, which includes also RPA1C and MUS81. It is possible that one of these genes or another not yet characterized could be responsible of the meiotic phenotypes of established and resynthesized lines [225]. In addition, the regions around the minor QTLs include *MSH3*, a gene involved in DNA repair [226], but it is not clear whether this gene may be considered a candidate [225].

#### 4.2.2. Arabidopsis

In the last decade, the *Arabidopsis* genus has risen as an excellent model for analyzing the consequences of WGD on meiosis [227]. This genus includes several polyploids of different ages and origins. In addition, the diploid progenitors of the polyploids are still found in nature. *Arabidopsis suecica* (2n = 4x = 26) and *A. kamchatica* (2n = 4x = 32) are allotetraploids, whereas *A. arenosa* and *A. lyrata* can be found in diploid (2n = 2x = 16) or tetraploid (2n = 4x = 32) populations.

*Arabidopsis suecica* and *A. kamchatica* present a diploid-like meiotic behavior and disomic inheritance [228]. However, *A. suecica* neotetraploids (obtained by the hybridization of autotetraploid *A. thaliana* and *A. arenosa*) display multivalents and reduced pollen viability [229]. A QTL named *BOY NAMED SUE* (*BYS*), together with other multiple genomic loci, seems to be involved in controlling homoeologous recombination in this allotetraploid species [229]. Furthermore, different chromosomal rearrangements might have contributed to the cytological diploidization [230]. A recent study has reported that *A. suecica* genome is colinear with the ancestral genomes of *A. thaliana* and *A. arenosa*, showing no subgenome dominance in expression and stable transposon dynamics, but with an upregulation of meiotic genes in the *A. thaliana* subgenome [231]. All these data suggest that even though the diploid progenitors of the *A. suecica* are quite divergent, a genetic control system should evolve to achieve meiotic diploidization.

*Arabidopsis arenosa* is an outcrossing species highly diverse that can be found in both diploid and tetraploid populations. Cytogenetic studies in tetraploids have revealed that most of homologous chromosomes associate randomly as bivalents during meiosis [232,233]. It has been hypothesized that a slower progression through prophase I could contribute to this diploidization [233]. In contrast, extensive multivalent formation and reduced fertility is observed in synthetic neotetraploids obtained from colchicine treated diploids [232]. On the other hand, *A. arenosa* diploid lines display a higher frequency of chiasmata per bivalent compared to the established autotetraploid [232]. These data support the idea that either the reduction of CO frequency or the increase of CO interference promote the formation of bivalents over multivalents, to achieve balanced chromosome segregation during meiosis in polyploids [234]. Indeed, CO interference, measured by localizing E3 ligase HEI10 foci, is strong in established autotetraploid plants of *A. arenosa*, but weak in synthetic neotetraploids of this species [235]. In this context, it is important to highlight that the reduction in chiasma frequency has also been observed in other established autotetraploid species [236]. However, there are species in which chiasma frequency increases by 75% over that in diploids [237]. To add more complexity, it is remarkable to note that some natural autotetraploids form multivalents with no substantial reduction in fertility compared to diploids [238].

Interestingly, signatures of selection found in meiotic genes of *A. arenosa* might be the consequence of genomic changes leading to genomic stability. These genes include elements related to the cohesin complex (*SMC3*, *REC8/SYN1*, and *PDS5*), components of the meiotic axes and synaptonemal complex (SC) (*ASY1*, *ASY3*, *ZYP1a*, and *ZYP1b*), and homologous recombination factors (*PRD3*) [232,239,240]. For this reason, it has been suggested that the diploid-like pairing of *A. arenosa* is the consequence of modifications in the structural components of the meiotic chromosomes. Specifically, for ASY1, a single amino-acid change within the HORMA protein domain was found at a very high frequency in tetraploid populations, whereas it was detected at a very low frequency in diploids [239]. The presence of this *ASY1* mutant allele in tetraploids is associated with a reduced formation of multivalents [241]. Likewise, in established autotetraploid plants of *A. lyrata*, the frequency of multivalents and chiasma distribution is associated with the segregation of an *ASY3* allele [242].

In the model species most commonly used for meiosis studies, *A. thaliana* (2n = 2x = 10), some natural tetraploid accessions have been found, but the cytogenetic studies performed have been scarce [243,244]. However, studies that focus on the analysis of colchicine-induced polyploids are more abundant [245,246,247] (Figure 1). Santos and coworkers [246] demonstrated that the high multivalents frequency observed in the first generation of *A. thaliana* autotetraploids decreases in successive generations of self-crossing, suggesting rapid adaptation to WGD. Remarkably, this cytological diploidization does not affect all chromosomes equally, since the small chromosomes suffer a more rapid decline of the frequency of multivalents. In a subsequent study, Parra-Nunez [248] reported that genetic differences between accessions have also an influence on chromosome associations during meiosis.

#### 4.2.3. *Solanum tuberosum*

Most cultivated potatoes, which represent the third most important food crop in the world, are autotetraploid (2n = 4x = 48) with an intriguing origin and evolution [249]. Despite the presence of four sets of homologous chromosomes, bivalents are commonly observed at metaphase I, although multivalent formation occurs sometimes [250]. The absence of preferences for pairing/synapsis and recombination between homologous chromosomes, together with outcrossing and a high level of heterozygosity, result in a large number of allelic combinations due to a polysomic pattern of inheritance [250].

It is important to establish the mechanism leading to bivalent formation in this autotetraploid to compare it with that responsible of the diploid-like meiotic behavior of allopolyploids. Cytogenetic studies have been difficult in potato due to the small size of chromosomes, but the use of bacterial artificial chromosomes (BACs) as probes in FISH experiments allowed the identification of individual chromosomes [251,252]. In an elegant study, He and coworkers [253] applied oligonucleotide-based painting probes to identify four different potato chromosomes. They demonstrated that the four homologous chromosomes form a SC quadrivalent configuration in 66–78% of pachytene meiocytes. However, cells with one chiasmate quadrivalent at metaphase I were reduced to 21–42%. The reduction in the frequency of quadrivalents as meiosis progresses has also been observed in wheat, and this mechanism (transformation of SC zygotene-pachytene quadrivalents into pairs of bivalents at metaphase I) has been proposed as a possible mechanism of diploidization of polyploid species [236,254].

Other studies have been focused on the comparison between diploid and tetraploid potato varieties. Remarkably, the CO frequency per bivalent (at least for some individual chromosomes) in certain tetraploid varieties was lower than in a diploid variety [255]. This observation concurs with the idea of reduction in the mean chiasma frequency per cell in the evolution of autotetraploid species [234]. Taking into account that in potato open chromatin regions, marked by H3K4me3, present a higher CO frequency [256], several epigenetic mechanisms, in addition to genetic factors, might be involved in the variation of this recombination landscape.

## 5. Readjustments of the Merged Genomes

### 5.1. Genomic Changes

Genomes from two diverged species that merge are usually unstable at early stages of allopolyploid formation and experience massive genetic changes including structural variation caused by deletions, inversions, translocations or homoeologous exchanges, together with epigenetic changes, such as transposable element (TE) activation and transcriptional gene silencing mediated by small RNAs, resulting in heritable loss of gene expression from previously active genes [8,257,258,259,260].

Genome sizes of polyploids are typically smaller than expected, suggesting that genome downsizing is a common readjustment in the diploidization process [261]. The loss of genetic material is a non-random process. It particularly affects to house-keeping genes or nuclear-encoded organellar genes [35,262], whereas genes involved in signal transduction and transcription are preferentially retained [263]. 

In wheat, allopolyploidization causes an immediate and non-random loss of both, coding and non-coding DNA sequences. Specifically, tetraploid wheat (*T. durum*) shows a reduction of 2–10% in the DNA content relative to the summed amounts of its diploid progenitors [264]. A similar situation occurs in the hexaploid wheat (*T. aestivum*) [265]. In this species, the rDNA loci of the A and D subgenomes have been lost in the evolution [265]. The DNA loss has contributed to increase the divergence between the homoeologous chromosomes, favoring its diploid-like meiotic behavior [174,264,266]. However, although many duplicated genes were lost, several copies of some of them are retained. Gene retention could serve as source of variation for natural selection to enhance possible adaptation to environmental changes [263]. In this context, the three homoeologous copies of most wheat meiotic genes are retained and show balanced expression to ensure proper meiotic progression [198]. There are, however, exceptions: the copy of the meiotic gene *SPO11-2* in subgenome A is not expressed, while its homoeologues in the other two subgenomes are functional [267]. Gene retention could also involve the acquisition of a novel expression domain or a new and beneficial role (neofunctionalization). This process has been reported for specific transcription factors, allowing the acquisition of different roles in regulatory development and plant morphology [268,269].

On the other hand, gene subfunctionalization involves the expression of duplicated genes in a tissue-specific way or in different developmental stages. It also ensures balanced expression among the different subgenomes [260,270]. The analysis of 727 RNAseq data sets in *T. aestivum* indicates that around 15–20% of genes present a tissue-specific differentiated homoeologous expression [271]. In the *Brassica* genus, the two paralogous genes *SHORT SUSPENSOR* (*SSP*) and *BRASSINOSTEROID KINASE 1* (*BSK1*) acquired different roles in the evolution. While *BSK1* retains its original role in hormonal transduction, *SSP* diverged to acquire a new function in zygote elongation by losing the kinase domain [272].

In addition to rearrangements affecting the copy number of genes, exons or small repeats, larger structural mutations resulting from reciprocal and non-reciprocal homoeologous exchanges abound in polyploids [273]. These rearrangements, involving small telomeric regions, intercalary segments of variable size, and even entire chromosome arms, alter the copy number of large genomic portions containing genic and non-genic DNA sequences, which is a representative feature of segmental allopolyploids. Extensive and repeated pattern of chromosomal variation has been reported in different populations of the natural allopolyploid *Tragopogon miscellus*, formed multiple times in the past 90 years [148]. Other segmental allopolyploids are quinoa, *Chenopodium quinoa* [274], tobacco, *Nicotiana tabacum* [275], *Brassica* [276,277], peanuts, *Arachis hypogaea* [278,279], and the synthetic allotetraploid rice obtained from the cross *O. sativa* subsp *indica* × *O. sativa* subsp *japonica* [280,281]. The complexity of chromosomal rearrangements can increase over generations, providing a very wide genomic diversity, on which natural selection can act promoting adaptation of neopolyploids. In fact, in *Brassica*, segmental allopolyploidy promoted phenotypic diversification of traits such as glucosinolate metabolism, flowering time or disease resistance [276].

Investigation of the constitution and evolution of subgenomes present in different allotetraploid plant species has shown that one subgenome, called dominant subgenome, tends to preserve more genes than the other subgenome. In addition, genes of the dominant subgenome tend to be more expressed than their homoeologous counterparts retained in the recessive subgenome [282]. Genome dominance involves events such as chromosome rearrangements of the types indicated above, which increase the dominant genome size instead of the submissive one, a preference in gene silencing of the submissive genome through epigenetic changes, or preference in activation of TEs from the dominant genome [270]. The occurrence of genome dominance normally appears following the hybridization process, throughout the first generations [283,284], or through multiple rounds of polyploidy [282]. In most cases, genome dominance manifests by upregulation or downregulation of the dominant or submissive genome, respectively [270,284,285]. The main elements responsible for this dominance would probably be trans–acting factors, that is, regulatory proteins such as transcription factors that operate through sequence-specific DNA-binding motifs. Dominance seems to arise in the genome with the most efficient factors [286]. For example, in the new synthetic allopolyploid *Cucumis sativus* × *C. hystryx* drastic changes at genomic level emerge rapidly after hybridization, while others occur in later generations at a slower rate. The study has revealed that the *C. sativum* subgenome is dominant, preserving more sequences and showing a higher expression level than the *C. hystryx* genome [287]. However, in tetraploid cotton, downregulation of the homoeologous gene copies of the submissive genome is mediated by both, cis- and trans-regulatory elements from the dominant genome [288]. Nevertheless, genome dominance is not present in all plant species. In *Oryza*, the short-term responses to genome merger are manifested in a high proportion of homoeologous gene pairs showing unequal expression [213].

### 5.2. Changes in the Activity of Transposable Elements (TEs)

Hybridization and polyploidization frequently trigger TE activation [269]. This has important consequences since TEs are the most abundant element in the genome of many plants. For example, in wheat, these elements represent about 85% of the genome [289].

TEs play a key role in plant evolution, since they are source of genetic diversity, allowing adaptation to new environments [290,291,292,293]. The repetitive nature of TEs offers numerous sequences scattered through the whole genome, which are potential sites for recombination and, therefore, represent a major source of chromosome rearrangements. TEs may represent a substrate for new genes and gene functions [294] and can provide promoters or transcriptional regulatory elements that change gene expression levels [295]. In addition, TEs transposition can create insertions and/or other mutations along the genome sequence, which might confer an adaptive advantage to new species [296]. Specifically, TEs seems to be involved in the adaptation to different stresses by modification of the expression of stress-related genes [297]. This phenomenon has been reported in the case of aluminum resistance genes [298] or in genes involved in the response to different diseases in pepper [299]. In wheat, Poretti and coworkers [300] showed how a specific class of TEs, Miniature Inverted-repeat Transposable Elements (MITEs), contribute to the regulation of neighboring genes via micro RNAs (miRNAs), increasing the immune response to the powdery mildew pathogen. In a meiotic context, TEs, providing sequence homology, are also involved in the modification of the recombination pattern along the chromosomes [301,302].

Hybridization and polyploidization not only allow the combination of two different TE populations, and siRNAs controlling them, but also affect the expression of flanking genes [295]. In addition to offer new regulatory sequences, TEs can also be the source of small RNAs that affect gene expression [260]. The reactivation of LTRs (Long Terminal Repeats) in resynthesized hexaploid wheat produces alterations in the expression of neighboring genes [303,304]. Similar results were observed in different species such as synthetic *Arabiposis* polyploids [22], synthetic *Cucumis* polyploids [287], or *Gossypium* [305], among others. TEs can also experience an increase in copy number following the hybridization, as it has been reported in tobacco [306] or *Brassica* [307]. Exceptions have been found, for example in *A. arenosa*, where polyploidization caused no change in the copy number [308].

### 5.3. Changes at the Gene Expression and Regulatory Level

The expression level of duplicated genes in allopolyploids, instead of being exclusively additive relative to that of progenitors, suffers a deep modification called “transcriptome shock” [309]. Gene expression changes, including genome dominance and non-additive expression patterns, have been reported in synthetic and natural polyploid species of various taxa, such as, *Arabidopsis* [310], *Tragopogon* [311], *Coffea* [312], *Gossypium* [288], *Oryza* [313], *Mimulus* [314] and *Triticum* [315,316]. The transcriptomic adjustment to the polyploid condition might take place either during the first generations, after the hybridization and/or WGD, or after a long term period [317]. For example, Zhao and coworkers [262], reported that among the homoeologous gene pairs of the A and B subgenomes, either from natural tetraploid wheat or extracted from hexaploid wheat, with differential expression relative to a synthetic tetraploid, most of them had only one differentially expressed copy, which was more often that of the B subgenome. In addition, differentially expressed genes were more abundant in the A and B extracted subgenomes than in those of the natural tetraploid wheat. This suggests that chromatin remodeling produced to adjust gene expression levels is an irreversible process that initiates at early generations and increases with the course of evolution. The non-additive expression pattern means that the expression level of a particular gene in a polyploid plant is not equal to the average of the gene expression levels in the two parents [318]. This has been observed in synthetic *Arabidopsis* allotetraploids (*A. thaliana* × *A. arenosa*) where around 6% of the genes differ in their expression level relative to the mid-parental value. Most of these genes were also differentially expressed among progenitors and non-additive gene regulation was derived from repression in 65% of genes [310].

As stated above, factors, such as TEs, genome dominance and cis- and trans-regulatory elements, contribute to gene expression regulation. In fact, the merge of two genomes could generate new forms of interactions between parental regulatory factors [286]. It has been demonstrated that the level of interactions between trans-factors of polyploids is about 54–64% higher than those found in diploids [319]. In wheat, polyploidization caused mainly elimination of redundant genes as well as appearance of inter-subgenome trans-regulation [262]. In addition, the merge of two different small RNA populations (such as miRNAs and siRNAs) can trigger the emergence of new regulatory mechanisms in allopolyploids [320,321]. Expression of these small RNAs can also be non-additively regulated [322]. In this context, a high siRNA density at genes associated with TEs has been reported to have a negative effect on gene expression of the D genome in nascent allohexaploid wheat [323].

However, modification of gene expression can be also influenced by epigenetic changes such as alterations in the pattern of methylation of histones and DNA [318]. Indeed, a study carried out on hexaploid wheat and its progenitor *Ae. tauschii* showed that modification of the methylation pattern is responsible of altered expression in 11% of genes [324]. Accordingly, changes of histone and DNA methylation are responsible of the Nucleolar Organizing Region (NOR) silencing in the subgenome A of a synthetic allotetraploid wheat, causing its further elimination [325]. Nucleolar dominance controlled by changes on methylation of DNA and histones has been also observed in the allopolyploid *A. suecica* (*A. thaliana* × *A. arenosa*), where the rRNA genes from *A. thaliana* are silenced while those from *A. arenosa*, are transcribed [259,326]. Likewise, DNA methylation changes observed in synthetic allopolyploid *B. napus* (AACC), affect mainly to silencing of genes of the C genome [327].

Epigenetic changes may also modulate the polyploid phenotype. For instance, *A. suecica* shows late flowering compared to its progenitors. In these species, the flowering time is controlled by two genes, the *FLOWERING LOCUS C* (*FLC*), that repress flowering, and *FRIGIDA* (*FRI*) that upregulates *FLC*. In *A. suecica* both *FLC* copies, from *A. thaliana* and *A. arenosa*, are upregulated by H3K4 trimethylation (H3K4me3) and H3K9 acetylation causing flowering delay [328]. The photoperiod in domesticated allotetraploid cotton (*G. barbadense* × *G. hirsutum*, AADD genomes) is also modified relative to the parental species. The *CONSTANT-LIKE 2* (*COL2*) gene is hypermethylated in both wild species, which is responsible of plant photoperiod sensitivity. During the domestication process, hypermethylation disappeared in *COL2* of the D genome, contributing to photoperiod insensitivity of the allotetraploid. This epigenetic change has allowed to produce cotton in different environments (such as subtropical) [329].

The merger and doubling of independent genomes profoundly impact their genetic architecture, the expression mode of merged genes, and the physiological machinery responsible of the allopolyploid phenotype. However, changes in genome organization, gene expression and molecular interactions do not occur only suddenly after hybridization but are cumulative throughout the polyploid evolution. Feldman and Levy [174] distinguished between revolutionary changes, that is, those arising in the early stages of allopolyploid formation and evolutionary changes, namely, changes produced more gradually over time. Understanding the impact that revolutionary and evolutionary changes have had on allopolyploid evolution is essential for a comprehensive knowledge of the dimension of the temporal progression needed to become a stable and well adapted allopolyploid species. As suggested by Nieto-Ferliner and coworkers [260] revolutionary changes most likely represent the tip of the iceberg as compared to later evolutionary innovations. Despite the advances produced in the last decades with the use of genomic tools for probing genomes and transcriptomes, the way by which polyploidization leads to phenotypic diversity and evolutionary diversification is poorly understood [260]. Consequently, it is also unknown how the different are the evolutionary patterns of allopolyploids relative to diploids.

## 6. Conclusions

Speciation by allopolyploidy is a complex evolutionary process that initiates with interspecific hybridization and is followed of the hybrid chromosome number duplication, cytological diploidization of the primitive allopolyploid and genetical diploidization derived from cumulative genomic changes over evolutionary timescale. Interspecific hybridization seems to be a relatively frequent phenomenon among plants conditioned by the reproductive system. Interpecific hybridization has been exploited in several research experiments for interspecific gene transfer. In addition, very valuable information on the genetic and epigenetic changes triggered by the genome merger has been obtained from synthetic hybrids and allopolyploids. WGD is also starting to be quantified and different cellular defects have been identified as responsible of the production of unreduced gametes. While the chromosome constitution of the hybrids may be responsible, at least in part, or the production of 2n gametes, there is evidence that the parental genotypes are also important to achieve WGD. On the other hand, there is accumulated evidence that meiotic and mitotic cell divisions are indeed highly vulnerable to environmental stress. Interspecific hybridization and WGD may facilitate genetic diversification and even provide an emergent saltational speciation as response to environment changes. The cytological diploidization depends on genetic systems evolved at the early allopolyploid stage that suppress recombination between homoeologous chromosomes. The locus *Ph1* in wheat is the best studied example, but the genetic basis of the diploid-like behavior is unknown in most allopolyploids. Understanding the mode of action of homoeologous recombination suppressors has also implication in their application for useful gene introgression into crops through meiotic recombination. To establish how the different are the mechanisms responsible of the cytological diploidization of autopolyploids and allopolyploids is also of great importance, since many autopolyploids show also preferential bivalent formation at meiosis. Genomic changes and transcriptomic modifications generated in the primitive allopolyploid can increase in complexity in the course of evolution giving rise to phenotypic innovations, which can be exposed to natural selection or drift. The advent of high-throughput molecular genetics and advances in DNA sequencing technologies provide experimental tools to investigate changes produced during the evolutionary trajectory of extant allopolyploid species and identify relevant genomics signatures of their cryptic long-term modifications capable of generating adaptation and speciation.
